# Ten rules for optimizing ventilatory settings and targets in post-cardiac arrest patients

**DOI:** 10.1186/s13054-022-04268-7

**Published:** 2022-12-17

**Authors:** Denise Battaglini, Paolo Pelosi, Chiara Robba

**Affiliations:** 1 Anesthesia and Intensive Care, San Martino Policlinico Hospital, IRCCS for Oncology and Neuroscience, Genoa, Italy; 2 Department of Surgical Sciences and Integrated Diagnostics, University of Genoa, Genoa, Italy

**Keywords:** Cardiac arrest, Mechanical ventilation, Lung-protective ventilation, Mechanical power, Brain injury

## Abstract

Cardiac arrest (CA) is a major cause of morbidity and mortality frequently associated with neurological and systemic involvement. Supportive therapeutic strategies such as mechanical ventilation, hemodynamic settings, and temperature management have been implemented in the last decade in post-CA patients, aiming at protecting both the brain and the lungs and preventing systemic complications. A lung-protective ventilator strategy is currently the standard of care among critically ill patients since it demonstrated beneficial effects on mortality, ventilator-free days, and other clinical outcomes. The role of protective and personalized mechanical ventilation setting in patients without acute respiratory distress syndrome and after CA is becoming more evident. The individual effect of different parameters of lung-protective ventilation, including mechanical power as well as the optimal oxygen and carbon dioxide targets, on clinical outcomes is a matter of debate in post-CA patients. The management of hemodynamics and temperature in post-CA patients represents critical steps for obtaining clinical improvement. The aim of this review is to summarize and discuss current evidence on how to optimize mechanical ventilation in post-CA patients. We will provide ten tips and key insights to apply a lung-protective ventilator strategy in post-CA patients, considering the interplay between the lungs and other systems and organs, including the brain.

## Background

Cardiac arrest (CA) is a major cause of morbidity and mortality with a high potential for detrimental systemic and cerebral complications [1]. Several therapeutic and supportive strategies have been implemented over the last years to optimize outcomes of post-CA patients, aiming at the improvement in neurological outcomes and survival [2, 3]. Among others, supportive strategies include appropriate settings of mechanical ventilation, aiming at optimizing gas exchange and limiting ventilator-induced lung injury (VILI), while avoiding systemic complications [3]. Mechanical ventilation should be targeted to limit hypoxemia and hyperoxemia and to maintain normal carbon dioxide levels [4–8], which are possible causes of secondary brain and reperfusion damage, lung damage, and poor survival [3, 9–16].

The literature in non-CA patients agrees on the importance of the use of lung-protective ventilator strategies (i.e., targeting at low tidal volume = *V*_T_ of 6–8 ml/Kg predicted body weight (PBW), low plateau pressure = *P*_PLAT_ < 20 cmH_2_O_,_ driving pressure = Δ*P* < 13 cmH_2_O and low positive end-expiratory pressure (PEEP) < 7 cmH_2_O [5, 17–27] with some safety measures for patients with/or at risk of brain injury [28]. Recently, the concept of mechanical power (MP), the mechanical energy delivered per time by the ventilator on the respiratory system or the lung, has also been proposed as an important component of mechanical ventilation settings. High MP was found associated with worse outcomes in non-acute respiratory distress syndrome (ARDS) [29] and ARDS patients [30], but this effect has not been completely elucidated in post-CA patients [28]. Additionally, the optimal ventilatory targets, i.e., oxygen and carbon dioxide levels in post-CA patients, still deserve to be clarified [3]. This review aims at discussing the current advances in mechanical ventilation strategies in patients with the post-CA syndrome. We propose ten key rules for optimizing mechanical ventilation in patients after CA, considering the interplay between the lungs and other systems and organs, including the brain.

## Rule one: tidal volume should be protective

*V*_T_ represents one of the key parameters of lung-protective ventilator strategies. Low tidal volume (*V*_T_) in patients without ARDS (*V*_T_ of 6–8 mL/kg PBW) resulted in no differences in ventilator-free days, intensive care unit (ICU), and hospital length of stay, 28-day and 90-day mortality in comparison with an intermediate *V*_T_ strategy [21]. In the PRoVENT-iMiC study, Pisani et al. found no differences in *V*_T_ between patients with higher or lower lung injury prediction score (around 8 mL/kg PBW) but lower values (around 7 mL/kg PBW) were applied in patients with ARDS [19]. A meta-analysis of 20 trials in critically ill patients without ARDS concluded that a strategy with lower *V*_T_ was associated with less pulmonary infection, atelectasis, and mortality [31]. The guidelines of the European Society of Intensive Care Medicine (ESICM) and the European Resuscitation Council (ERC) provided some insights for mechanical ventilation (MV) in brain-injured and post-CA patients, suggesting applying a *V*_T_ of 6–8 ml/kg PBW, but these recommendations were based on other populations and only small evidence in post-CA patients [32]. Few studies investigated the role of *V*_T_ in CA patients. In an observational study, Sutherasan et al. reported the use of a median *V*_T_ of 8.9 mL/kg PBW with a median PEEP of 3.5 cmH_2_O [18]. A *V*_T_ higher than 10 mL/kg PBW was associated with ICU-acquired pneumonia [18]. In another study from Beitler et al., the use of lower *V*_T_ was independently associated with favorable neurocognitive outcome, and ventilator-free days in a cohort of 256 post-CA patients [33]. A sub-analysis of the Target Temperature Management (TTM)1 trial reported a median *V*_T_ of 7.7. mL/kg PBW (60% of patients were ventilated with a *V*_T_ lower than 8 mL/kg PBW) and *V*_T_ was not associated with mortality [26]. Evidence from a study including three different cohorts of patients with neurological diseases from distinct years (2004, 2010, and 2016) suggested an implementation over years of lung-protective ventilator strategies, especially regarding *V*_T_ [34]. A very recent sub-study of the TTM2 trial including 1848 post-CA patients reported a median *V*_T_ of 7 mL/kg PBW and a PEEP of 7 cmH_2_O without differences between survivors and non-survivors [28]. All these studies suggest a progressive reduction in *V*_T_ over years which can be interpreted as an expression of increasingly application of lung-protective ventilator strategies in patients with CA. The association between lower *V*_T_ and outcome is not consistent across studies, and no randomized controlled trials have been performed in the specific subgroup of patients [28]. Nevertheless, in other clinical settings, guidelines recommend using *V*_T_ between 6 and 8 mL/kg PBW. Volume-controlled ventilation is currently more frequently used compared to pressure-controlled ventilation [18], but no data suggest any superiority between the different modes. The use of assisted ventilation, mainly pressure support ventilation (PSV), is increasingly used in patients after CA [18]. We suggest that in post-CA patients the *V*_T_ should be set between 6 and 8 mL/kg PBW, in volume- or pressure-controlled ventilation but keeping in mind the interplay between *V*_T_ and other parameters of MV (i.e., *P*_PLAT,_ Δ*P*, PEEP, MP) as well as hemodynamics. Assisted ventilation may be used according to clinical conditions and the level of sedation of the patient.

## Rule two: plateau pressure should be personalized

*P*_PLAT_ is another important parameter of lung-protective ventilation, since it depends on the relationship between volume and compliance of the respiratory system in the absence of flow. Maintenance of *P*_PLAT_ below 20 cmH_2_O is recommended in patients without ARDS to reduce mortality [23, 35]. The PREVENT trial in critically ill patients without ARDS reported lower *P*_PLAT_ (*P*_PLAT_ = 18 cmH_2_O) in the group at lower *V*_T_ as compared to intermediate *V*_T_ (*P*_PLAT_ = 21 cmH_2_O), without significant differences in ventilator-free days, length of stay, complications, and mortality between the two groups [21]. The Relax trial found similar *P*_PLAT_ between the group at lower PEEP = 5 cmH_2_O (19.9 cmH_2_O) and that at higher PEEP = 8 cmH_2_O (20 cmH_2_O), therefore suggesting that a lower PEEP strategy could preferable in patients without ARDS [22]. In a study in post-CA patients, *P*_PLAT_ significantly differed among three cohorts of patients at different timeframes, with the highest value observed in the year 1998 and the lowest in 2010 (22.7 and 19.5 cmH_2_O, respectively), suggesting a progressive temporal change in ventilator setting in this population [18]; in addition, *P*_PLAT_ higher than 17 cmH_2_O was found to be associated with ARDS development [18]. A very recent study in a cohort of patients post-CA suggested the adoption of *P*_PLAT_ < 20 cmH_2_O [28]. As for *V*_T_, *P*_PLAT_ should be set with the aim to reduce VILI, but most importantly should be considered within a personalized ventilatory strategy, accounting also for other ventilatory parameters which can be associated with mortality, including respiratory rate, driving pressure, and MP [28]. In the study by Robba et al., *P*_PLAT_ was significantly associated with 6-month mortality [28]. The *P*_PLAT_ of the respiratory system and transpulmonary pressure might differ in case of marked alterations of the chest wall elastance, as occurs in obesity. In obese patients or those with increased intra-abdominal pressure with *P*_PLAT_ > 27 cmH_2_O, a simplified formula may help estimate the required correction of *P*_PLAT_: *P*_PLAT_ target + (intra-abdominal pressure-13 cmH_2_O)/2 [36]. In mechanically ventilated non-obese patients, the average intra-abdominal pressure is 13 cmH_2_O and half of the intra-abdominal pressure is transmitted to the thoracic cavity [37]. We suggest that in post-CA patients the *P*_PLAT_ should be kept equal or lower than 20 cmH_2_O and corrected for intra-abdominal pressure when clinically indicated.

## Rule three: positive end-expiratory pressure should be low but enough

PEEP represents a key component of mechanical ventilation. Evidence agrees on that very low PEEP or zero PEEP can aggravate the risk of atelectasis and worsen lung damage [38]. The effects of higher PEEP on brain injury and intracranial pressure have been widely studied but still result a matter of debate [39]. High PEEP can increase intrathoracic pressure, potentially impairing left ventricle activity, decreasing the preload, afterload, and contractility, thus reducing venous return, which reflects on jugular veins acting as Starling resistors from intrathoracic pressure to the brain. Jugular veins present a valvular mechanism that limits the transmission of excessive intrathoracic pressure to the brain compartment. Indeed, it seems that if PEEP is lower than venous pressure, an excessive increase in pressure to the brain does not occur [40]. In the PRoVENT trial, when Δ*P* was included in the model, PEEP resulted associated with outcome [23]. In the Relax trial including critically ill patients with uninjured lungs, a lower PEEP strategy resulted in similar mortality and ventilator-free days than a higher PEEP strategy without the occurrence of severe hypoxemia [22]. The PRoVENT-iMiC observational study concluded that PEEP values were not associated with outcomes at multivariable analysis [19]. Few studies investigated the practice of the use of PEEP in post-CA population. Sutherasan et al. reported a mean PEEP value of 3.5 cmH_2_O in three cohorts of post-CA patients [18], while in a sub-analysis of the TTM1 trial, the mean PEEP was 6 cmH_2_O [26]. No randomized trials exist about PEEP in post-CA, but meta-analysis of trials in critically ill patients without ARDS confirmed that a higher PEEP strategy (5–30 cmH_2_O) was not associated with a better outcome, although it can decrease the incidence of ARDS and hypoxemia [41]. In this context, zero or very low PEEP levels should be avoided in the post-CA population to guarantee optimal oxygenation while limiting atelectasis or dynamic hyperinflation, hemodynamic derangement, impaired brain physiology, and other systemic complications [42, 43]. The most recent sub-analysis of the TTM2 trial in post-CA patients suggested that in this population, the application of a lung-protective ventilator strategy is even more common, including the use of a PEEP higher than in the past. In this study, a median value of 7 cmH_2_O of PEEP was used, but PEEP alone was not associated with patients’ outcomes, suggesting that a combination of ventilator parameters should be accounted for setting the ventilator in post-CA patients (i.e., Δ*P* which is a function of *P*_PLAT_ and PEEP) [28]. We suggest that in post-CA patients a PEEP of 5 cmH_2_O should be initially used to reach a SatO_2_ at least above 92% and progressively increase in case of oxygen desaturation or worsening of respiratory mechanics.

## Rule four: pay attention to the driving pressure!

**Fig. 1:**
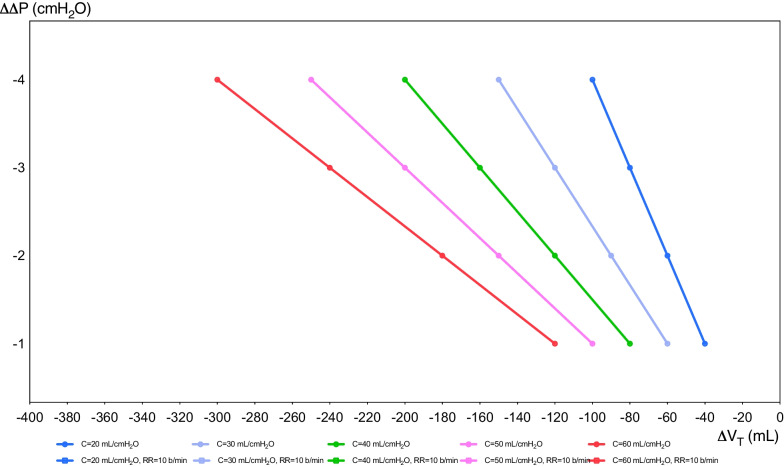
Variation of tidal volume in function of variation of driving pressure. In this figure, we reported variation of Δ*V*_T_ in function of the variation of Δ*P* (ΔΔ*P*) at different static compliances of the respiratory system (20, 30, 40, 50, 60 mL/cmH_2_O). The respiratory rate varies according to the formula of Costa et al. Basal *V*_T_ is assumed to be 7 ml/kg of predicted body weight (70 kg)

**Fig. 2:**
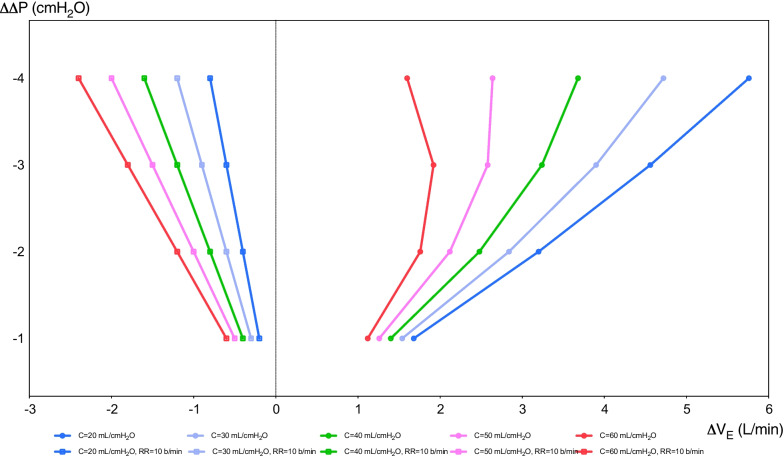
Variation of minute ventilation in function of variation of driving pressure. In this figure, the minute ventilation (*V*_E_) varies in function of the variation of driving pressure Δ*P* (ΔΔ*P*) with (1) fixed respiratory rate to 10 breaths/min on the left, and (2) variable respiratory rate according to the formula by Costa et al. on the right. Basal *V*_E_ is assumed to be 7 ml/kg of predicted body weight (70 kg) multiplied by 10 breaths/minute = 4.9 L/min

Driving pressure (Δ*P*) represents the distending pressure of the lungs, being the result of *P*_PLAT_ minus PEEP, and representing the stress applied to the respiratory system. The *V*_T_ changes differently affect the variation of Δ*P* (ΔΔ*P*) and *P*_PLAT_, in relation to different static compliance of the respiratory system (Fig. 1). The PRoVENT study in patients without ARDS concluded that Δ*P* was not independently associated with in-hospital mortality, although Δ*P* value was available for 343 patients only [23]. In the PRoVENT-iMiC, median Δ*P* was similar in patients with or without lung injury but was higher in those with ARDS. At multivariable analysis, Δ*P* resulted not to be associated with outcome [19]. In post-CA patients, in a sub-analysis of the TTM1 trial, a median Δ*P* = 14.7 cmH_2_O was reported [18]. A Δ*P* < 13 cmH_2_O was suggested, since higher values are independently associated with higher mortality [21, 22, 28, 32]. In the study of Robba et al., Δ*P* was independently associated with mortality and poor neurological outcome. The formula of Costa et al., [(4 × Δ*P*) + respiratory rate], previously investigated in patients with ARDS, was recently applied by Robba et al. in a large cohort of post-CA patients, showing a stronger association with poor neurological outcome and mortality, when compared to MP alone [28]. This formula can easily guide ventilator settings at the bedside. Reducing *V*_T_ to lower Δ*P* by 1 cmH_2_O is worthwhile only if the PaCO_2_ can be kept constant by increasing the respiratory rate by less than 4 breaths/min. Conversely, it may be worthwhile reducing the respiratory rate by four breaths if the increase in *V*_T_ needed to maintain a constant PaCO_2_ results in an increase in Δ*P* less than 1 cmH_2_O. The variation of Δ*P* (ΔΔ*P*) as a function of the variation of minute ventilation (Δ*V*_E_) at different static compliances of the respiratory system is depicted in Fig. 2. On the right side, the respiratory rate varies according to the Δ*P*, while in the left side, the respiratory rate is kept fixed at 10 breaths/min. We suggest in post-CA patients to maintain a Δ*P* < 13 cmH_2_O optimizing the *V*_T_ for the respective compliance of the respiratory system.

## Rule five: respiratory rate should be targeted to pHa and PaCO_2_

Respiratory rate is one of the key variables of mechanical ventilation. Its contribution as harm during MV has been often underestimated. However, both respiratory rate per se and respiratory rate insensitivity (meaning as the ability of respiratory rate to control the minute ventilation) may become injurious during MV, thus promoting VILI, dynamic hyperinflation, and respiratory alkalosis [44]. Additionally, respiratory rate represents a major drive of chemical feedback such as arterial partial pressure of oxygen (PaO_2_), arterial partial pressure of carbon dioxide (PaCO_2_) and pHa. This assumes a pivotal relevance in patients who have/ or are at risk of brain injury such as post-CA patients, in order to modulate cerebral blood flow and vascular tone, as high PaCO_2_ can cause cerebrovascular vasodilation, and cerebral edema [45]. In normal conditions, when either volume or pressure increases, the respiratory rate decreases via the Hering–Breuer reflex [44]. The regulation of PaCO_2_ and oxygenation in post-CA population is challenging but deserves important attention in order to avoid secondary brain damage [28]. In a study by Harmon et al. respiratory rate, none of the other ventilation parameters was independently associated with 28-day mortality [26]. Similarly, in the study by Robba et al., the median respiratory rate was 17 breaths/min and resulted independently associated with 6 months of poor neurological outcome and mortality. As discussed above, respiratory rate should be adapted to Δ*P* and total mechanical power. We suggest that in post-CA patients, the respiratory rate should be kept in a range between 8 and 16 breaths/min.

## Rule six: mechanical power is an attractive target, but with caution

**Fig. 3:**
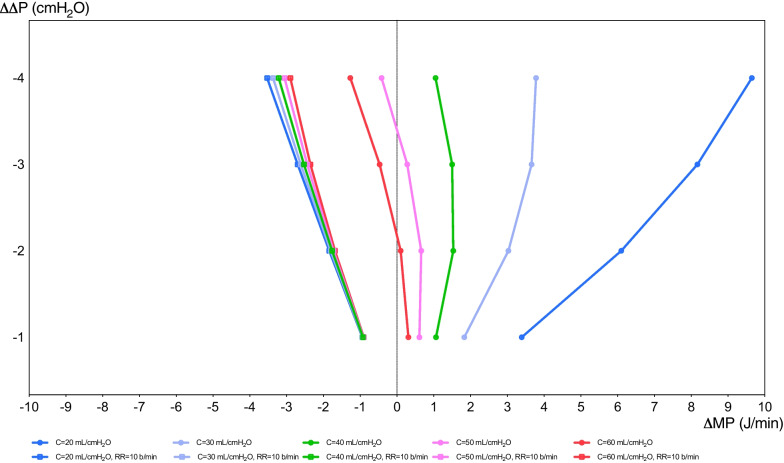
Variation of mechanical power in function of variation of driving pressure. In this figure, the variation of mechanical power (ΔMP) in J/min changes in function of the variation of driving pressure Δ*P* (ΔΔ*P*) with (1) fixed respiratory rate to 10 breaths/min on the left, and (2) variable respiratory rate according to the formula by Costa et al. on the right. The figure depicts the variation at different compliances of the respiratory system (20, 30, 40, 50, 60 mL/cmH_2_O). Basal MP is assumed to be calculated on a tidal volume (*V*_T_) of 7 ml/kg of predicted body weight (70 kg) and PEEP = 0 cmH_2_O, according to the formula of MP in pressure control ventilation = 0.098 × respiratory rate × *V*_T_ × (PEEP + Δ*P*)

MP is the product of mechanical energy and respiratory rate applied to the respiratory system or the lungs. MP accounts for several parameters of MV, and for this reason in the current years is gaining increased attention as a possible determinant of patient outcome [46]. In an observational cohort of critically ill patients without ARDS, MP resulted associated with in-hospital mortality, ICU mortality, 30-day mortality, ventilator-free days, ICU and hospital length of stay, with a consistent increase in the risk of death with MP higher than 17.0 J/min [47]. MP was even more strongly associated with mortality when it was normalized by body mass index [29]. The MP equation is made up of several parameters of lung-protective ventilation. The respective role of each individual ventilatory setting parameter within MP to determine effects on VILI and clinical outcomes is not clear. In a recent study of ARDS patients, Costa et al. [46] found that the impact of the Δ*P* on mortality was four times as large as that of the respiratory rate. This suggests that a reduction of 1 cmH_2_O in Δ*P* should be associated with a maximum increase in respiratory rate of 4 breaths/min, while a reduction of 3 cmH_2_O in Δ*P* to a maximum increase in respiratory rate of 12 breaths/min. However, these relationships might change at different levels of lung injury and respiratory or lung elastances. Previous studies on post-CA patients did not assess MP as a possible variable associated with outcome. In 2022, Robba et al. found that in post-CA patients, MP was independently associated with both 6 months of mortality and neurological outcome [28]. Figure 3 provides a theoretical analysis of five different respiratory system compliances (from 20 to 60 mL/cmH_2_O) during variations of Δ*P* (ΔΔ*P*) in the function of variation of MP (ΔMP). In the right part of the graph, the formula of Costa et al. [(4 × Δ*P*) + respiratory rate] is adopted to see the variation of MP at different respiratory system compliances and maximum accepted different respiratory rates. In the left part of the graph, the respiratory rate is maintained constant to 10 breaths/minute while the respiratory system compliance is changing. Especially at lower respiratory system compliances, the change in MP is higher at the highest respiratory rate permitted by Costa et al. formula [(4 × Δ*P*) + respiratory rate]. This suggests that respiratory rate should be controlled within certain limits and play a relevant role to determine an excessive increase in MP, especially when the respiratory compliance is low. This formula can easily drive the MV setting at the bedside, when manipulating respiratory rate for keeping constant pHa (around 7.25) and PaCO_2_ [28]. In post-CA patients, only few of the parameters which constitute the MP formula were independently associated with poor outcome and VILI, while others were not (i.e., *V*_T_ and PEEP) [28]. This suggests that caution is required to set the ventilator settings only on MP and conclusive evidence on its real clinical utility at the bedside is warranted especially in post-CA patients. As per evidence to date, if assessed at the bedside, we suggest that in post-CA patients MP should be targeted as lower than 17 J/min, taking into account Δ*P* and respiratory rate [28, 47]

## Rule seven: oxygenation should be accurately targeted to normoxia

Oxygenation is a key parameter to monitor in post-CA patients since this syndrome can activate different mechanisms such as reperfusion injury and oxidative stress, which can contribute to brain injury and neuronal damage [48]. Both hypoxemia and hyperoxemia are identified as possible detrimental stimuli on the outcome of critically ill patients. Hypoxemia in post-CA syndrome acts by altering the cerebral aerobic metabolism, which, if not restored, can lead to neuronal injury and cell death. Once the oxygen is restored after the return of spontaneous circulation, a possible reperfusion mechanism occurs, thus further accelerating neuronal death [3]. On the contrary, hyperoxemia may lead to the increased production of reactive oxygen species in the mitochondria and oxidative damage to the brain cells and seems to influence patients’ outcomes as well [3, 49]. The threshold responsible for hypoxic neuronal damages has yet to be defined, and, till now, it has been generally set at 60 mmHg [50–53]. Harmon et al. reported a liberal approach for oxygenation, thus the patients presenting with PaO_2_ up to 100 mmHg and with a high fraction of inspired oxygen levels. In non-survivors, oxygenation was lower [26]. However, other reports suggested that hypoxia can be detrimental also below 100 mmHg [18], while Ebner et al. suggested that either hypoxemia or hyperoxemia was not associated with outcome in post-CA patients, but recommended to titrate peripheral saturation of oxygen (SpO_2_) between 94 and 98% [5]. A very recent study demonstrated that a value of 60 mmHg could underestimate the risk of hypoxemia in the post-CA population as the best lower threshold associated with increased mortality was found at PaO_2_ of 69 mmHg, while the best upper threshold of PaO_2_ was 195 mmHg [54]. Regarding hyperoxia, a recent meta-analysis [55], showed that severe hyperoxemia (PaO_2_ > 300 mmHg) was associated with poor neurological outcome and mortality at follow-up in post-CA patients. In a study by Roberts et al., hyperoxemia with values > 300 mmHg was associated with poor outcome [56]. In the study of Robba et al., both hyperoxemia “per se” and the dose (AUC) of hyperoxemia over time were associated with mortality but not with poor neurological outcome. This suggests that the effect of hyperoxemia may depend on the duration of the exposure to high oxygen levels. In this study, the best upper threshold of PaO_2_ was > 195 mmHg. This is different from previous studies, but may explain why in previous studies a threshold of 300 mmHg was not associated with mortality or neurological outcome [57–60]. Current evidence also suggests that the “time passed” in one or another condition may play a key role in patient outcome, more than the single value per se, and that more attention should be paid to the titration of oxygen to lower levels than those applied in the past. The ICU-ROX trial confirmed no significant impact of conservative oxygen therapy (target peripheral saturation of oxygen = SpO_2_ > 90% and < 97%) as compared with usual oxygen therapy (SpO_2_ without upper limits but higher than 90%) on ventilator-free days [61]. Significant recommendations will come from the Mega-ROX trial (ANZCTRN 12620000391976) that will compare a liberal (SpO_2_ without upper limits, but > 90%) versus conservative (SpO_2_ 91–94%) oxygen therapy in critically ill patients [62]. In a recent randomized controlled trial, targeting a restrictive (68–75 mmHg) versus a liberal (98–105 mmHg) oxygenation strategy in comatose patients after CA did not change the outcome (death, severe disability, and coma) [63]. While waiting for the results of ongoing clinical trials, according to the findings to date, a cutoff of PaO_2_ of 70–110 mmHg seems reasonable in this patient population.

## Rule eight: carbon dioxide should be within normal ranges: so far

The role of carbon dioxide levels is frequently underestimated in post-CA patients. Hypercapnia and hypocapnia are detrimental to the brain physiology. An alteration of PaCO_2_ can widely affect the changes in intracellular pH and influence metabolic energy and oxygen demand also to the brain [3]. Hypocapnia is responsible for cerebral vasoconstriction and ischemic injury shifting to anaerobic metabolism and activating a systemic inflammatory response. On the other hand, hypercapnia can cause vasodilatation and promote a decrease in cerebral blood flow [3]. Although some researchers suggested the use of mild hypercapnia (PaCO_2_ 50–55 mmHg) to improve cerebral oxygenation in comparison with normocapnia, elevated levels of PaCO_2_ can lead to lower pH and negatively influence outcome [64]. Therefore, changes in PaCO_2_ as well as mechanical ventilation during and after CA can affect carbon dioxide and pH levels and trigger dangerous pathways around pH, cellular demand, and catecholamine release [3], thus affecting the outcome. Ebner et al. [25] did not find any association between hypocapnia (< 34 mmHg) or hypercapnia (> 45 mmHg) and neurological outcome post-CA. While waiting for the results of the TAME randomized clinical trial (NCT03114033), the appropriate threshold to apply in post-CA patients is yet to be defined. According to the literature, a value of PaCO_2_ ranging between 35 and 50 mmHg seems to be preferable [3].

## Rule nine: temperature can influence ventilatory function

The role of temperature management in post-CA patients is becoming increasingly clear, but its effect on the setting of the ventilatory parameter and gas exchange is still uncertain. Hypothermia at a targeted temperature of 33 °C did not confer a benefit as compared with a targeted temperature of 36 °C [65]. Targeted hypothermia at 33 °C followed by controlled rewarming did not reduce 6-month mortality as compared to targeted normothermia [66]. Temperature is a potential influencer of gas exchange, and the solubility of PaCO_2_ increases at lower temperatures. Indeed, blood gas analysis is commonly normalized to temperature, otherwise can report incorrect values. Similarly, in a small retrospective study, the PaCO_2_ decreased significantly more in the target temperature management group as compared with controls [67]. Regarding oxygen, a recent study found no significant differences in thresholds of oxygen related to the mortality between hypothermia and normothermia targets, thus suggesting that hypothermia does not improve oxygen tolerance [66]. The effect of temperature is relatively slight if dead space is not increased. These effects of temperature were investigated through the equation of alveolar dead space ventilation: (PaCO_2_ – end-tidal (et)CO_2_)/PaCO_2_, where temperature management at 33 °C resulted in lower etCO_2_ levels and higher alveolar dead space fraction compared to 36 °C with similar minute ventilation [26]. This effect can be explained by the lower pulmonary perfusion due to increased vasoconstriction, being also in accordance with the higher lactate levels at 33 °C compared to 36 °C [26]. In patients who remain comatose post-CA, the guidelines recommend continuous monitoring of core temperature and prevention of fever (defined as a temperature > 37.7 °C) for at least 72 h. Evidence is insufficient to recommend for or against temperature control at 32–36 °C or early cooling after CA [68].

## Rule ten: hemodynamics should be maintained stable

Hemodynamics represents another important piece for optimization of MV. In post-CA patients, MV, fluids and vasopressor management, and temperature control can influence hemodynamics and outcome. Positive pressure ventilation can affect pulmonary blood flow and distribution, thus worsening cardiovascular function and gas exchange [69]. Indeed, the ventilator should be set to allow adequate expiratory time to limit the development of dynamic hyperinflation, and the effects of intrinsic PEEP and positive MV pressures to avoid cardiovascular collapse, especially in post-CA patients who frequently report altered cardiovascular function [69]. The administration of fluids to restore the end-diastolic volume can be considered in presence of PEEP if concomitant impaired left ventricular contractility and cardiac output occur. Vasopressors, inotropes, or inodilators such as epinephrine, dobutamine, or milrinone can be used to support cardiac output after the optimization of fluids and ventilator setting, but they can increase oxygen demand. [69]. A very recent trial suggested that targeting a mean arterial pressure of 63 mmHg or 77 mmHg in post-CA patients does not improve mortality or neurological outcome [70]. These results suggested adopting an individualized hemodynamic strategy. The temperature target can impact hemodynamics. Hypothermia at 33 °C, when compared to 36 °C, was associated with hemodynamic alterations (i.e., decreased heart rate, elevated lactate levels, and need for increased vasopressor support). Low mean arterial pressure and the need for high doses of vasopressors were independently associated with increased mortality in both groups [71]. A post hoc analysis of the TTM2 trial found that in post-CA patients with moderate vasopressor support on admission, hypothermia at 33 °C group increased non-neurological death. Indeed in the 33 °C group, hemodynamic instability and arrhythmias were more frequent [72]. In summary, patients with post-CA syndrome need to be strictly monitored for possible detrimental respiratory and cardiovascular interactions, thus accounting for targeted temperature management (around 36 °C) and personalized cardiovascular targets.

## Future directions

**Fig. 4:**
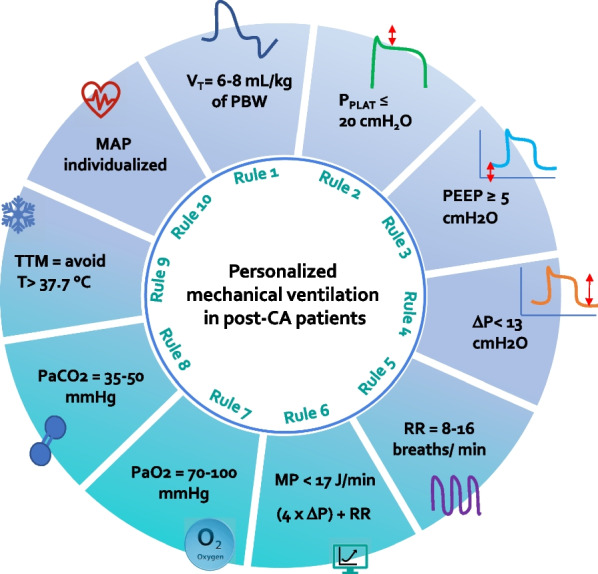
Ten key rules for optimizing ventilator setting in post-CA patients according to an organ protective mechanical ventilation strategy. *V*_T_ = tidal volume, PBW = predicted body weight, PEEP = positive end-expiratory pressure, RR = respiratory rate, Δ*P* = driving pressure, MP = mechanical power, PaO_2_ = arterial partial pressure of oxygen, PaCO_2_ = arterial partial pressure of carbon dioxide, TTM = target temperature management, MAP = mean arterial pressure, CA, cardiac arrest

The role of protective mechanical ventilation in post-CA patients is becoming even more clear. Figure 4 resumes the key rules for optimizing the setting of the ventilator in post-CA patients while accounting for lungs–heart and brain interactions. The PRoVENT-iMiC study showed that protective mechanical ventilation is easy to achieve following simple rules also in low- and middle-income countries where the resources are scarcer than high-income counties [19]. However, how to properly set the ventilator of a patient with post-CA syndrome as well as ARDS or other diseases needs education and training with specialized programs of intervention [73].

## Conclusions

The role of protective and personalized mechanical ventilation setting in patients without ARDS and after CA is becoming more evident. Optimization of mechanical ventilation is cheap and may be adopted in high and middle-low economic income countries requiring only training and education. However, the individual role of each parameter of protective ventilation to minimize lung injury and their association with clinical major outcomes have not been completely elucidated in post-CA patients and deserve further research.

## Data Availability

Not applicable.

## Abbreviations

Δ*P*: Driving pressure
ARDS: Acute respiratory distress syndrome
CA: Cardiac arrest
ERC: European Resuscitation Council
ICU: Intensive care unit
MP: Mechanical power
MV: Mechanical ventilation
PaCO_2_: Arterial partial pressure of carbon dioxide
PaO_2_: Arterial partial pressure of oxygen
PBW: Predicted body weight
PEEP: Positive end-expiratory pressure
*P*_PLAT_: Plateau pressure
SpO_2_: Peripheral saturation of oxygen
TTM: Target temperature management
*V*_E_: Minute ventilation
VILI: Ventilator-induced lung injury
*V*_T_: Tidal volume
